# Sex- and age-specific associations of *VEGFA* polymorphisms with multiple sclerosis susceptibility

**DOI:** 10.1080/07853890.2026.2715843

**Published:** 2026-08-22

**Authors:** Kriste Kaikaryte, Simona Rubene, Alvita Vilkeviciute-Petraite, Renata Balnyte, Ingrida Uloziene, Rasa Liutkeviciene

**Affiliations:** ^a^ Laboratory of Ophthalmology, Neuroscience Institute, Medical Academy, Lithuanian University of Health Sciences, Kaunas, Lithuania; ^b^ Medical Academy, Lithuanian University of Health Sciences, Kaunas, Lithuania; ^c^ Department of Neurology, Medical Academy, Lithuanian University of Health Sciences, Kaunas, Lithuania; ^d^ Department of Otorhinolaryngology, Lithuanian University of Health Sciences, Kaunas, Lithuania; ^e^ Department of Ophthalmology, Medical Academy, Lithuanian University of Health Sciences, Kaunas, Lithuania

**Keywords:** Multiple sclerosis, VEGFA, VEGFA polymorphism, single nucleotide polymorphism, Haplotype analysis, Lithuanian population, clinical phenotype

## Abstract

**Aim:**

To evaluate six *VEGFA* polymorphisms (rs1570360, rs699947, rs3025033, rs2146323, rs1413711 and rs833061) and serum *VEGFA* concentrations in 270 Lithuanian patients with multiple sclerosis (MS) and 270 matched healthy controls.

**Methods:**

Genotyping was performed using real-time PCR, and serum *VEGFA* levels were measured by enzyme-linked immunosorbent assay. Statistical analyses were conducted using IBM SPSS version 31.0.

**Results:**

Nominal differences in *VEGFA* genotype and allele distributions were observed in sex- and age-stratified analyses. Among females, the rs1413711 C allele was more frequent (*p* = 0.005), whereas the rs833061 C allele was less frequent (*p* = 0.006), in MS patients than controls. In participants aged >38 years, the rs1413711 C allele was also more frequent in MS cases (57.5% vs. 46.0%, *p* = 0.008). Logistic regression identified nominal associations, particularly for rs1413711, rs699947 and rs833061, but none remained significant after correction for multiple testing. Serum *VEGFA* levels were higher in MS patients than controls (*p* = 0.004). Nominal genotype-related differences in serum *VEGFA* levels and haplotype associations with reduced MS odds were also observed, but did not remain significant after multiple-testing correction. No consistent associations were found between* VEGFA* variants and clinical parameters.

**Conclusion:**

*VEGFA* genetic variation and elevated serum *VEGFA* may be associated with MS, but the genetic findings require confirmation in larger independent cohorts.

## 1. Introduction

Multiple sclerosis (MS) is an immune-mediated, inflammatory, demyelinating and neurodegenerative disorder of the central nervous system (CNS) [1,2]. It is recognized as the most common autoimmune disease affecting this system. MS impacts approximately 2.8 to 2.9 million people worldwide, with prevalence increasing globally [3–5]. According to a systematic review, higher rates of MS are generally reported in women and in populations residing at higher latitudes, such as Northern Europe, North America and parts of Asia [6,7]. In Lithuania, the incidence of multiple sclerosis has significantly increased over the period from 2001 to 2015, according to a 2018 study. The average rate during these years was 6.5 cases per 100.000 people, with expectations that this figure would rise to 13 per 100.000 by 2020. Together with its Baltic neighbours, Lithuania (population approximately 2.9 million) remains a region with a relatively high MS incidence compared to other countries. More than 2.000 individuals in Lithuania have been diagnosed with MS. The study also highlighted that women are 1.5 to 2 times more susceptible to MS than men in this population [8]. MS is typically diagnosed between the ages of 20 and 40 and significantly reduces quality of life by causing non-traumatic neurological disability in young individuals [9]. The pathophysiology and aetiology of MS are complex, involving a combination of genetic and environmental factors that trigger an autoimmune response [10]. Environmental contributors include infectious agents (e.g. Epstein–Barr virus), nutritional deficiencies (e.g. vitamin D), smoking [11–13] and latitude [1]. Genetic studies indicate that MS is not directly inherited; however, a family history of MS modestly increases risk [14,15]. Since the initial Genome-Wide Linkage Analysis by Ebers et al. in 1996, no single main causative gene has been identified [16]. To this day, over 200 genes have been linked to increased susceptibility, most notably within the *HLA* region, with HLA-DRB1*15 being the strongest genetic risk factor [17]. MS-associated genetic variants can be divided into three main categories: common variants (prevalence > 5%) that confer a small increased risk, low-frequency variants (prevalence < 5%) with moderate effects and rare variants, which have minor effects and are more difficult to detect and characterize. Genome-Wide Association Studies (GWAS) remain the preferred approach for identifying common variants [14]. In this study, we focus on the human vascular endothelial growth factor A (*VEGFA*) to explore the potential association between *VEGFA* gene polymorphisms (rs1570360, rs699947, rs3025033, rs2146323, rs1413711 and rs833061) and serum VEGFA levels in patients with multiple sclerosis in Lithuania.

The *VEGFA* gene is located on chromosome 6. VEGFA, a member of the VEGF family, plays a crucial role in vascular permeability, angiogenesis and endothelial cell growth and migration [18]. Since MS affects the brain and spinal cord, its progression is marked by damage to the blood-brain barrier (BBB), which results in increased vascular permeability and promotes angiogenesis [19]. Therefore, VEGFA is considered a key factor in the advancement of MS pathology [20,21]. Its involvement in MS pathogenesis is mainly associated with angiogenesis, BBB disruption and neuroinflammation. The BBB normally protects the CNS from harmful circulating agents [22–24]. However, in early MS, inflammatory BBB dysfunction permits infiltration of pathogenic immune cells (including T and B lymphocytes, immunoglobulin G and cytokines) into the CNS. These activated lymphocytes trigger an autoimmune response, resulting in myelin and axonal damage [25–27]. VEGFA increases vascular permeability, compromising the BBB and facilitating immune cell entry, which leads to inflammation and tissue injury [28]. Mechanistically, VEGFA-induced permeability is primarily mediated through VEGFR2 signalling, which activates downstream pathways including ERK1/2, nitric oxide production and platelet-activating factor synthesis, as well as PLCβ2-dependent calcium fluxes and endothelial junctional remodelling [29]. Additionally, VEGFA promotes angiogenesis in response to hypoxia and inflammation within MS lesions. Activated T cells, particularly those undergoing Th1 polarization, represent an important source of VEGFA, producing it in response to antigenic stimulation, interleukin-2 and hypoxic conditions. These cells further amplify pro-inflammatory responses through interferon-gamma (IFN-γ) while suppressing regulatory cytokines such as IL-10 [30]. Immune cells such as T cells and macrophages secrete VEGFA, stimulating new vessel formation and extracellular matrix remodeling, thereby sustaining inflammation and tissue damage. Under hypoxic conditions, which are commonly observed in active MS lesions, VEGFA expression is upregulated through hypoxia-inducible factor-1α (HIF-1α), which binds to the VEGFA promoter and enhances its transcription. This response promotes adaptive angiogenesis and vascular permeability aimed at restoring oxygen supply, but may simultaneously exacerbate BBB disruption and neuroinflammation [31]. VEGFA expression also correlates with elevated inflammatory mediators, including hypoxia-inducible factor 1 (HIF-1), tumour necrosis factor-alpha (TNFα) and interferon-gamma (IFNγ), which further amplify VEGFA-driven angiogenesis and inflammation, creating a feedback loop that exacerbates disease progression [32,33]. It should also be noted that disease-modifying therapies used in multiple sclerosis, including monoclonal antibodies such as Natalizumab and anti-CD20 therapies (e.g. Ocrelizumab and Rituximab), may influence inflammatory pathways and could potentially modulate VEGFA expression and circulating levels [34]. VEGFA involvement in MS pathogenesis underscores the importance of investigating its polymorphisms and serum levels within the Lithuanian population. Given that MS primarily affects the brain and spinal cord, with disease progression marked by BBB disruption, increased vascular permeability and pathological angiogenesis, it is possible that *VEGFA* variants may play a critical role in driving MS progression.

## 2. Materials and methods

### 2.1. Subjects and ethical statement

The study was conducted in accordance with the Declaration of Helsinki and was approved by the Kaunas Regional Biomedical Research Ethics Committee, Lithuanian University of Health Sciences, Kaunas, Lithuania (Approval No. BE-2-137). Written informed consent was obtained from all participants prior to inclusion in the study. A total of 540 individuals were enrolled in the study, which took place at the Laboratory of Ophthalmology at the Neuroscience Institute, Lithuanian University of Health Sciences. The study population was divided into two groups, as follows.

#### 2.1.1. Group I: Multiple sclerosis patients (*n* = 270)

This group comprised 270 patients diagnosed with multiple sclerosis who received treatment at Lithuanian University of Health Sciences clinics in Kaunas from January 1, 2020, to August 1, 2025. Inclusion was restricted to patients with a confirmed MS diagnosis according to the 2017 revised McDonald criteria [35]. Upon diagnosis, patients underwent lumbar puncture, and cerebrospinal fluid (CSF) was analyzed. The presence of intrathecal oligoclonal bands (OCBs) was assessed using isoelectric focusing combined with IgG-specific immunofixation techniques. Demographic variables (age, gender) and clinical characteristics (age at MS diagnosis, MS disease course and duration, presence of pyramidal, sensory, brainstem, cerebellar, pelvic and visual symptoms and disability assessed using the EDSS score) were recorded for all patients. Disability was evaluated by the Kurtzke expanded disability status scale (EDSS) [36]. Magnetic resonance imaging (MRI) data were also collected. The clinical information was retrospectively extracted from outpatient medical records. Patients with systemic illnesses such as diabetes mellitus, malignancy, connective tissue disorders, chronic infections, autoimmune diseases or those who had undergone tissue or organ transplantation were excluded to avoid potential confounding factors. Clinical MS subtypes (e.g. RRMS and SPMS) were recorded for all patients. However, due to the limited number of patients in progressive MS subgroups, particularly SPMS, subtype-specific analyses were not performed. Instead, analyses were conducted using the combined MS cohort to ensure sufficient statistical power and avoid unreliable estimates from small subgroup sizes.

Detailed treatment data were not included in the present analysis, as the primary focus of the study was on genetic polymorphisms and serum VEGFA levels.

#### 2.1.2. Group II: Control group (*n* = 270)

The control cohort included 270 healthy individuals matched by age and sex to the MS group. These participants were free of any personal history of autoimmune or neurological disorders.

The study cohort and recruitment procedures have been described previously in a study investigating *IL-33* genetic variants and circulating IL-33 levels in the same Lithuanian multiple sclerosis population [37]. The present study uses the same ethically approved cohort but addresses a distinct research objective by evaluating *VEGFA* polymorphisms and serum VEGFA concentrations.

### 2.2. Polymorphism selection

The study concentrated on six polymorphisms within the *VEGFA* gene: rs1570360, rs699947, rs3025033, rs2146323, rs1413711 and rs833061. Although these variants have not been conclusively associated with MS, previous studies suggest links to diseases related to MS, including autoimmune and neurodegenerative disorders. The polymorphisms were selected because they reside in regulatory regions of the gene: rs1570360, rs699947 and rs833061 are located in the promoter near the transcription start site, influencing transcription factor binding and gene expression [38]. The other SNPs: rs3025033, rs2146323 and rs1413711 are positioned within intronic regions, which might affect processes such as alternative splicing or other regulatory mechanisms, though their functional impact is less defined [39]. This combination allowed exploration of both transcriptional and post-transcriptional regulatory effects that may contribute to *VEGFA* involvement in MS susceptibility. Participants were further stratified by age using the median age of the study population (38 years), resulting in two groups (≤38 years and >38 years). This approach was applied to ensure balanced subgroup sizes and adequate statistical power for exploratory subgroup analyses. The selected cut-off falls within the commonly reported peak diagnostic age range for multiple sclerosis (20–40 years), although it does not represent a biologically defined threshold [40].

### 2.3. DNA extraction, *VEGFA* genotyping and VEGFA serum-level determination

DNA isolation and genotyping of polymorphisms rs1570360, rs699947, rs3025033, rs2146323, rs1413711 and rs833061 were carried out at the Ophthalmology Laboratory, Neuroscience Institute (Lithuanian University of Health Sciences). Genomic DNA was extracted from venous blood through a salting-out procedure. Briefly, white blood cells were suspended in a buffer, followed by cell lysis using detergents. Proteinase K digestion eliminated protein contaminants, after which chloroform treatment was applied to remove residual proteins. Finally, DNA was precipitated by adding ethanol.

Genotyping utilized TaqMan^®^ SNP Genotyping assays (Thermo Scientific, Pleasanton, CA, USA). The assays were processed on a Step One Plus real-time PCR instrument (Applied Biosystems, Foster City, CA, USA) per manufacturer’s protocols. Reaction mixtures prepared for a 96-well plate contained 1.5 μL of DNA sample and 8.5 μL of PCR mix, including negative controls. The Allelic Discrimination mode was employed for genotype determination based on fluorescent VIC and FAM reporter dyes as specified by the assay provider.

Serum VEGFA concentrations were determined in 40 MS patients and 40 controls using the Human VEGF-A ELISA kit (Thermo Fisher Scientific, Cat. No. BMS277-2). Serum samples were divided into 100 μL aliquots and stored at −80 °C until testing. ELISA assays were run in duplicate according to the manufacturer’s instructions. Absorbance readings were taken at 450 nm using a Multiskan FC microplate photometer (Thermo Scientific, Waltham, MA, USA). VEGFA levels were calculated using a standard curve generated from seven recombinant human VEGF-A concentrations. The assay sensitivity was 7.9 pg/mL.

### 2.4. Statistical analysis

Data analyses were performed using IBM SPSS Statistics software version 31.0 (IBM Corp., Armonk, NY, USA). Categorical variables such as sex distribution were summarized as frequencies and percentages and compared by chi-square (χ^2^) tests. Variables with non-normal distribution, including age and serum VEGFA levels, were reported as medians with interquartile ranges (IQR) and compared between groups using the Mann-Whitney U test.

Genotype and allele frequencies of the six *VEGFA* polymorphisms were compared between MS patients and controls using the χ^2^ test. To evaluate the association between genotypes and MS susceptibility, binary logistic regression analyses were performed. Odds ratios (ORs) with 95% confidence intervals (CIs) were calculated. The most appropriate genetic model (dominant, recessive, codominant) was selected using the lowest Akaike information criterion (AIC) value.

An a priori power calculation was performed for the overall and stratified analyses assuming a minor allele frequency of approximately 0.30 and an odds ratio in the range of 1.3–1.5. Given that stratified analyses by sex and age reduce effective sample size and statistical power, a hierarchical analysis strategy was applied, with the overall analysis performed first, followed by pre-specified subgroup analyses.

Haplotype analyses were conducted *via* SNPStats software (available at https://www.snpstats.net/snpstats/, accessed January 15, 2025) [41]. Linkage disequilibrium between SNPs was assessed through D’ and r^2^ statistics. Logistic regression was used to determine associations between *VEGFA* haplotypes and MS risk, with ORs and 95% CIs reported. Haplotypes with a frequency below 1% were combined into a “rare” category to enable robust analysis. Two-sided p-values less than 0.05 were considered statistically significant. Figures and graphs were prepared using GraphPad Prism version 9.0.0 for Mac (GraphPad Software, San Diego, CA, USA), accessed February 21, 2025.

To control for multiple testing, Bonferroni correction was applied separately for primary genetic association analyses. For single nucleotide polymorphism (SNP) association analyses, including logistic regression models, the significance threshold was adjusted according to the total number of SNP – model comparisons performed (six SNPs across five genetic models; adjusted significance threshold *p* < 0.0017).

For haplotype analysis, Bonferroni correction was applied according to the number of haplotypes evaluated. Analyses of serum VEGFA concentrations were considered exploratory due to the smaller sample size and were therefore interpreted based on nominal p-values, with appropriate caution.

## 3. Results

This study included 540 participants split evenly into two groups: 270 multiple sclerosis patients and 270 age- and gender-matched controls. The MS group consisted of 64.1% females (*n* = 173) and 35.9% males (*n* = 97), while the control group had 64.4% females (*n* = 174) and 35.6% males (*n* = 96). The median age of the participants was 38 years for the MS group and 39 years for the control group. Demographic details are shown in Table 1.

**Table 1.** Demographic characteristics of the study groups.

Characteristic	Group			ELISA group		p-value
	Control group n = 270 n (%)	Multiple sclerosis n = 270 n (%)	p-value	Control group n = 40 n (%)	Multiple sclerosis n = 40 n (%)	
Gender						
Males	96 (35.6)	97 (35.9)	0.928	20 (51.3) 19 (48.70	17 (42.5) 23 (57.5)	0.434
Females	174 (64.4)	173 (64.1)				
Age, years						
Median (IQR)	39 (24)	38 (15)	0.982	32 (21)	33.5 (22)	0.988

p-value – significance level.

Hardy–Weinberg equilibrium was evaluated in the control group for all investigated VEGFA polymorphisms. Genotype distributions of rs699947, rs2146323, rs1413711 and rs833061 were consistent with Hardy–Weinberg equilibrium, whereas rs1570360 and rs3025033 showed deviations from equilibrium (supplementary material Table S1).

### 3.1. Distribution of *VEGFA* (rs1570360, rs699947, rs3025033, rs2146323, rs1413711, rs833061) genotypes and alleles among multiple sclerosis patients and controls

Analysis of *VEGFA* gene variants (rs1570360, rs699947, rs3025033, rs2146323, rs1413711 and rs833061) showed no statistically significant differences in genotype or allele frequencies between MS patients and controls after applying the strict Bonferroni correction (Table 2).

**Table 2.** Distribution of *VEGFA* rs1570360, rs699947, rs3025033, rs2146323, rs1413711 and rs833061 genotypes and alleles in multiple sclerosis patients and control groups.

Genotype/Allele	Control group	Multiple sclerosis	p-value
	n = 270	n = 270	
	n (%)	n (%)	
***VEGFA rs1570360***			
GG	120 (44.4)	117 (43.3)	0.104
AG	96 (35.6)	115 (42.6)	** **
AA	54 (20.0)	38 (14.1)	
G	336 (62.2)	349 (54.6)	0.675
A	204 (37.8)	191 (35.4)	
***VEGFA rs699947***			
AA	85 (31.5)	66 (24.4)	0.113
AC	127 (47.0)	130 (48.1)	
CC	58 (21.5)	74 (27.4)	
A	297 (55.0)	262 (48.5)	0.033
C	243 (45.0)	278 (51.5)	
***VEGFA rs3025033***			
AA	179 (66.3)	167 (61.9)	
AG	75 (27.8)	95 (35.2)	
GG	16 (5.9)	8 (3.0)	
A	433 (80.2)	429 (79.4)	0.762
G	107 (19.8)	111 (20.6)	
***VEGFA rs2146323***			
CC	109 (40.4)	139 (51.5)	0.033
AC	118 (43.7)	94 (34.8)	** **
AA	43 (15.9)	37 (13.7)	
C	336 (62.2)	372 (68.9)	0.021
A	204 (37.8)	168 (31.1)	
***VEGFA rs1413711***			
TT	91 (33.7)	64 (23.7)	0.034
CT	120 (44.4)	134 (49.6)	** **
CC	59 (21.9)	72 (26.7)	
T	302 (55.9)	262 (48.5)	0.015
C	238 (44.1)	278 (51.5)	
***VEGFA rs833061***			
CC	91 (33.7)	67 (24.8)	0.063
CT	120 (44.4)	130 (48.1)	** **
TT	59 (21.9)	73 (27.0)	
C	302 (55.9)	264 (48.9)	0.021
T	238 (44.1)	276 (51.1)	

p-value – significance level and Bonferroni corrected the significance level when *p* = 0.05/30; the bolded results indicate significant differences between the groups.

Binary logistic regression analysis was conducted to assess the SNPs as potential risk factors for multiple sclerosis development; however, none of the analyzed variants showed statistically significant associations (Table 3).

**Table 3.** Binary logistic regression analysis of *VEGFA* rs1570360, rs699947, rs3025033, rs2146323, rs1413711 and rs833061 in multiple sclerosis patients and control groups.

Model	Genotype/allele	OR (95% CI)*	p-value	AIC
**Patients with multiple sclerosis**				
***VEGFA rs1570360***				
Co-dominant	AG vs. GG	1.229 (0.847–1.782)	0.278	748.051
	AA vs. GG	0.722 (0.444–1.174)	0.189	
Dominant	AG+AA vs. GG	1.046 (0.745–1.470)	0.795	750.531
Recessive	AA vs. AG+GG	0.655 (0.416–1.032)	0.068	749.231
Overdominant	AG vs. AA+GG	1.345 (0.951–1.902)	0.094	747.788
Additive	A	0.914 (0.726–1.151)	0.445	750.016
***VEGFA rs699947***				
Co-dominant	AC vs. AA	1.318 (0.880–1.975)	0.180	748.223
	CC vs. AA	1.643 (1.026–2.631)	0.039	
Dominant	AC+CC vs. AA	1.420 (0.973–2.073)	0.069	757.273
Recessive	CC vs. AC+AA	1.380 (0.930–2.048)	0.110	748.027
Overdominant	AC vs. AA+CC	1.046 (0.746–1.466)	0.796	750.532
Additive	C	1.283 (1.014–1.623)	0.038	746.249
***VEGFA rs3025033***				
Co-dominant	AG vs. AA	1.358 (0.939–1.963)	0.104	747.106
	GG vs. AA	0.536 (0.224–1.285)	0.162	
Dominant	AG+GG vs. AA	1.213 (0.853–1.725)	0.282	749.440
Recessive	GG vs. AG+AA	0.485 (0.204–1.152)	0.485	747.757
Overdominant	AG vs. AA+GG	1.411 (0.980–2.033)	0.064	747.159
Additive	G	1.046 (0.780–1.403)	0.764	750.509
***VEGFA rs2146323***				
Co-dominant	AC vs. CC	0.625 (0.432–0.904)	0.012	745.788
	AA vs. CC	0.675 (0.407–1.119)	0.128	
Dominant	AC+AA vs. CC	0.638 (0.454–0.897)	0.010	743.873
Recessive	AA vs. AC+CC	0.838 (0.521–1.349)	0.468	750.070
Overdominant	AC vs. AA+CC	0.688 (0.486–0.974)	0.035	746.118
Additive	A	0.769 (0.606–0.976)	0.031	745.884
***VEGFA rs1413711***				
Co-dominant	CT vs. TT	1.588 (1.060–2.377)	0.025	745.807
	CC vs. TT	1.735 (1.085–2.776)	0.021	
Dominant	CT+CC vs. TT	1.636 (1.122–2.386)	0.011	743.977
Recessive	CC vs. CT+TT	1.300 (0.876–1.930)	0.192	748.893
Overdominant	CT vs. CC+TT	1.232 (0.878–1.728)	0.228	749.141
Additive	C	1.327 (1.049–1.677)	0.018	744.965
***VEGFA rs833061***				
Co-dominant	CT vs. CC	1.471 (0.985–2.198)	0.059	747.051
	TT vs. CC	1.680 (1.054–2.678)	0.029	
Dominant	CT+TT vs. CC	1.540 (1.060–2.239)	0.024	745.430
Recessive	TT vs. CT+CC	1.325 (0.893–1.966)	0.162	748.631
Overdominant	CT vs. TT+CC	1.161 (0.827–1.628)	0.388	749.854
Additive	T	1.303 (1.033–1.645)	0.026	745.584

OR – odds ratio; AIC – Akaike information criteria; CI – confidence interval; p-value – significance level; Bonferroni corrected significance level when *p* = 0.05/30; the bolded results indicate significant differences between the groups.

### 3.2. Distribution of *VEGFA* (rs1570360, rs699947, rs3025033, rs2146323, rs1413711, rs833061) genotypes and alleles among multiple sclerosis patients and controls by gender

The distribution of *VEGFA* genotypes and alleles in female MS patients and controls is presented in Table 4. Nominal differences in genotype distribution were observed for rs699947 (*p* = 0.028), rs1413711 (*p* = 0.022) and rs833061 (*p* = 0.024), with a higher frequency of the homozygous variant genotypes (CC or TT) in MS patients compared to controls. Similarly, allele frequency analysis showed nominal differences for rs699947 (*p* = 0.009), rs1413711 (*p* = 0.005) and rs833061 (*p* = 0.006), with the minor alleles (C or T) more frequently observed in the MS group. No statistically significant differences were observed for rs1570360, rs3025033 or rs2146323 at either the genotype or allele level. However, after applying Bonferroni correction for multiple testing (adjusted significance threshold *p* < 0.0017), none of the observed associations remained statistically significant and should therefore be interpreted with caution.

**Table 4.** Distribution of *VEGFA* rs1570360, rs699947, rs3025033, rs2146323, rs1413711 and rs833061 genotypes and alleles across females in multiple sclerosis patients and control groups.

Genotype/Allele	Control group	Multiple sclerosis	p-value
	n = 174	n = 173	
	n (%)	n (%)	
***Females***			
***VEGFA rs1570360***			
GG	77 (44.3)	82 (47.4)	0.088
AG	62 (35.6)	71 (41.0)	** **
AA	35 (20.1)	20 (11.6)	
G	216 (62.1)	235 (67.9)	0.106
A	132 (37.9)	111 (32.1)	
***VEGFA rs699947***			
AA	54 (31.0)	41 (23.7)	0.028
AC	85 (48.9)	76 (43.9)	
CC	35 (20.1)	56 (32.4)	
A	193 (55.5)	158 (45.7)	0.009
C	155 (44.5)	188 (54.3)	
***VEGFA rs3025033***			
AA	117 (67.2)	100 (57.8)	0.110
AG	48 (27.6)	66 (38.2)	** **
GG	9 (5.2)	7 (4)	
A	282 (81.0)	266 (76.9)	0.179
G	66 (19.0)	80 (23.1)	
***VEGFA rs2146323***			
CC	69 (39.7)	85 (49.1)	0.154
AC	78 (44.8)	61 (35.3)	** **
AA	27 (15.5)	27 (15.6)	
C	216 (62.1)	231 (66.8)	0.197
A	132 (37.9)	115 (33.2)	
***VEGFA rs1413711***			
TT	58 (33.3)	42 (24.3)	0.022
CT	81 (46.6)	75 (43.4)	** **
CC	35 (20.1)	56 (32.4)	
T	197 (56.6)	159 (46.0)	**0.005**
C	151 (43.4)	187 (54.0)	
***VEGFA rs833061***			
CC	57 (32.8)	42 (24.3)	0.024
CT	82 (47.1)	75 (43.4)	** **
TT	35 (20.1)	56 (32.4)	
C	196 (56.3)	159 (46.0)	**0.006**
T	152 (43.7)	187 (54.0)	

p-value – significance level and Bonferroni corrected the significance level when *p* = 0.05/30; the bolded results indicate significant differences between the groups.

In male participants, no statistically significant differences were found in the genotype or allele frequencies of *VEGFA* polymorphisms (rs1570360, rs699947, rs3025033, rs2146323, rs1413711 and rs833061) between MS patients and controls (Table 5).

**Table 5.** Distribution of *VEGFA* rs1570360, rs699947, rs3025033, rs2146323, rs1413711 and rs833061 genotypes and alleles across males in multiple sclerosis patients and control groups.

Genotype/Allele	Control group	Multiple sclerosis	p-value
	n = 96	n = 97	
	n (%)	n (%)	
***Males***			
***VEGFA rs1570360***			
GG	43 (44.8)	35 (36.1)	0.346
AG	34 (35.4)	44 (45.4)	** **
AA	19 (19.8)	18 (18.6)	
G	120 (62.5)	114 (58.8)	0.452
A	35 (37.5)	80 (41.2)	
***VEGFA rs699947***			
AA	31 (32.3)	25 (25.8)	0.253
AC	42 (34.8)	54 (55.7)	
CC	23 (24.0)	18 (18.6)	
A	104 (54.2)	104 (53.6)	0.912
C	88 (45.8)	90 (46.4)	
***VEGFA rs3025033***			
AA	62 (64.6)	67 (69.1)	0.093
AG	27 (28.1)	29 (29.9)	** **
GG	7 (7.3)	1 (1.0)	
A	151 (78.6)	163 (84.0)	0.175
G	41 (21.4)	31 (16.0)	
***VEGFA rs2146323***			
CC	40 (41.7)	54 (55.7)	0.126
AC	40 (41.7)	59 (60.8)	** **
AA	16 (16.7)	16 (16.5)	
C	120 (62.5)	103 (53.1)	0.033
A	72 (37.5)	91 (46.9)	
***VEGFA rs1413711***			
TT	33 (34.4)	22 (22.7)	0.019
CT	39 (40.6)	75 (43.4)	** **
CC	24 (25.0)	56 (32.4)	
T	105 (54.7)	159 (46.0)	0.753
C	87 (45.3)	187 (54.0)	
***VEGFA rs833061***			
CC	34 (35.4)	25 (25.8)	0.059
CT	38 (39.6)	55 (56.7)	** **
TT	24 (25.0)	17 (17.5)	
C	106 (55.2)	105 (54.1)	0.831
T	86 (44.8)	89 (45.9)	

p-value – significance level and Bonferroni corrected the significance level when *p* = 0.05/30; the bolded results indicate significant differences between the groups.

Binary logistic regression analysis of *VEGFA* polymorphisms in females is presented in Table 6. Several nominal associations were observed. In particular, rs699947 showed increased odds of MS under the co-dominant (CC vs. AA: OR = 2.107, *p* = 0.013), recessive (OR = 1.901, *p* = 0.010) and additive models (OR = 1.447, *p* = 0.013). Similarly, rs1413711 and rs833061 demonstrated nominal associations under multiple models, including co-dominant, recessive and additive models (p-values ranging from 0.007 to 0.010). For rs3025033, nominal associations were observed in the co-dominant (AG vs. AA: OR = 1.609, *p* = 0.042) and overdominant models (*p* = 0.037). A protective effect was suggested for rs1570360 under the recessive model (OR = 0.519, *p* = 0.031). No significant associations were observed for rs2146323. However, after applying Bonferroni correction for multiple testing (adjusted significance threshold *p* < 0.0017), none of these associations remained statistically significant and should therefore be interpreted with caution.

**Table 6.** Binary logistic regression analysis of *VEGFA* rs1570360, rs699947, rs3025033, rs2146323, rs1413711 and rs833061 across females in multiple sclerosis patients and control groups.

Model	Genotype/allele	OR (95% CI)*	p-value	AIC
**Females**				
***VEGFA rs1570360***				
Co-dominant	AG vs. GG	1.735 (1.047–2.875)	0.758	480.134
	AA vs. GG	0.537 (0.285–1.009)	0.053	
Dominant	AG+AA vs. GG	0.881 (0.577–1.344)	0.557	482.695
Recessive	AA vs. AG+GG	0.519 (0.286–0.942)	0.031	478.229
Overdominant	AG vs. AA+GG	1.257 (0.815–1.940)	0.300	481.967
Additive	A	0.800 (0.598–1.071)	0.134	480.782
***VEGFA rs699947***				
Co-dominant	AC vs. AA	1.178 (0.707–1.962)	0.530	477.866
	CC vs. AA	2.107 (1.173–3.786)	0.013	
Dominant	AC+CC vs. AA	1.449 (0.901–2.330)	0.126	480.688
Recessive	CC vs. AC+AA	1.901 (1.166–3.099)	0.010	476.261
Overdominant	AC vs. AA+CC	0.820 (0.538–1.252)	0.358	482.197
Additive	C	1.447 (1.080–1.939)	0.013	476.794
***VEGFA rs3025033***				
Co-dominant	AG vs. AA	1.609 (1.018–2.543)	0.042 0.857	480.606
	GG vs. AA	0.910 (0.327–2.532)		
Dominant	AG+GG vs. AA	1.498 (0.968–2.320)	0.070	479.737
Recessive	GG vs. AG+AA	0.773 (0.281–2.125)	0.618	482.790
Overdominant	AG vs. AA+GG	1.619 (1.030–2.545)	0.037	478.639
Additive	G	1.282 (0.889–1.849)	0.183	481.253
***VEGFA rs2146323***				
Co-dominant	AC vs. CC	0.635 (0.400–1.007)	0.054	481.294
	AA vs. CC	0.812 (0.436–1.510)	0.510	
Dominant	AC+AA vs. CC	0.680 (0.445–1.041)	0.076	479.879
Recessive	AA vs. AC+CC	1.007 (0.563–1.799)	0.982	483.041
Overdominant	AC vs. AA+CC	0.670 (0.435–1.032)	0.070	479.728
Additive	A	0.833 (0.621–1.118)	0.224	481.558
***VEGFA rs1413711***				
Co-dominant	CT vs. TT	1.279 (0.771–2.121)	0.341	477.352
	CC vs. TT	2.210 (1.237–3.945)	**0.007**	
Dominant	CT+CC vs. TT	1.560 (10.975–2.493)	0.063	479.562
Recessive	CC vs. CT+TT	1.901 (1.166–3.099)	0.010	476.261
Overdominant	CT vs. CC+TT	0.879 (0.575–1.342)	0.549	482.682
Additive	C	1.481 (1.109–1.978)	0.008	475.829
***VEGFA rs833061***				
Co-dominant	CT vs. CC	1.241 (0.748–2.061)	0.403	477.560
	TT vs. CC	2.171 (1.215–3.881)	0.009	
Dominant	CT+TT vs. CC	1.520 (0.950–2.432)	0.081	479.972
Recessive	TT vs. CT+CC	1.901 (1.166–3.099)	0.010	476.261
Overdominant	CT vs. TT+CC	0.859 (0.562–1.311)	0.480	482.542
Additive	T	1.468 (1.098–1.961)	0.009	474.181

OR – odds ratio; AIC – Akaike information criteria; CI – confidence interval; p-value – significance level; Bonferroni corrected significance level when *p* = 0.05/30; the bolded results indicate significant differences between the groups.

In males (Table 7), no consistent associations between VEGFA polymorphisms and MS risk were identified. Although some nominal associations were observed – for example, rs1413711 in the overdominant model (OR = 2.269, *p* = 0.005) and rs833061 in the co-dominant and overdominant models (*p* = 0.045 and *p* = 0.018, respectively) – these did not remain significant after correction for multiple testing. Overall, the observed associations are likely influenced by multiple testing and should be considered exploratory.

**Table 7.** Binary logistic regression analysis of *VEGFA* rs1570360, rs699947, rs3025033, rs2146323, rs1413711 and rs833061 across males in multiple sclerosis patients and control groups.

Model	Genotype/allele	OR (95% CI)*	p-value	AIC
**Males**				
***VEGFA rs1570360***				
Co-dominant	AG vs. GG	1.590 (0.845–2.991)	0.150	269.420
	AA vs. GG	1.164 (0.531–2.549)	0.704	
Dominant	AG+AA vs. GG	1.437 (0.807–2.561)	0.218	269.028
Recessive	AA vs. AG+GG	0.923 (0.451–1.891)	0.828	269.502
Overdominant	AG vs. AA+GG	1.514 (0.849–2.700)	0.160	267.564
Additive	A	1.146 (0.783–1.677)	0.485	269.060
***VEGFA rs699947***				
Co-dominant	AC vs. AA	1.594 (0.821–3.095)	0.168 0.942	268.796
	CC vs. AA	0.970 (0.431–2.184)		
Dominant	AC+CC vs. AA	1.374 (0.736–2.565)	0.319	268.553
Recessive	CC vs. AC+AA	0.723 (0.361–1.448)	0.360	268.707
Overdominant	AC vs. AA+CC	1.615 (0.915–2.851)	0.099	266.801
Additive	C	1.023 (0.685–1.526)	0.912	269.538
***VEGFA rs3025033***				
Co-dominant	AG vs. AA	0.994 (0.531–1.862)	0.985 0.062	266.227
	GG vs. AA	0.132 (0.016–1.105)		
Dominant	AG+GG vs. AA	0.817 (0.448–1.488)	0.508	269.111
Recessive	GG vs. AG+AA	0.132 (0.016–1.098)	0.061	264.228
Overdominant	AG vs. AA+GG	1.090 (0.585–2.030)	0.786	269.476
Additive	G	0.710 (0.427–1.181)	0.187	267.781
***VEGFA rs2146323***				
Co-dominant	AC vs. CC AA vs. CC	0.611 (0.330–1.132) 0.463 (0.190–1.127)	0.117 0.090	267.392
Dominant	AC+AA vs. CC	0.569 (0.322–1.006)	0.052	265.750
Recessive	AA vs. AC+CC	0.575 (0.247–1.340)	0.200	267.864
Overdominant	AC vs. AA+CC	0.722 (0.403–1.294)	0.274	268.349
Additive	A	0.660 (0.438–0.994)	0.047	265.500
***VEGFA rs1413711***				
Co-dominant	CT vs. TT	2.269 (1.156–4.454)	0.017	263.619
	CC vs. TT	1.000 (0.435–2.996)	1.000	
Dominant	CT+CC vs. TT	1.786 (0.946–3.370)	0.074	266.295
Recessive	CC vs. CT+TT	0.593 (0.292–1.203)	0.147	267.414
Overdominant	CT vs. CC+TT	2.269 (1.275–4.038)	**0.005**	261.619
Additive	C	1.068 (0.712–1.601)	0.751	269.449
***VEGFA rs833061***				
Co-dominant	CT vs. CC	1.968 (1.016–3.814)	0.045	265.850
	TT vs. CC	0.963 (0.429–2.161)	0.928	
Dominant	CT+TT vs. CC	1.579 (0.851–2.930)	0.147	267.430
Recessive	TT vs. CT+CC	0.638 (0.317–1.281)	0.206	267.933
Overdominant	CT vs. TT+CC	1.999 (1.127–3.546)	0.018	263.859
Additive	T	1.044 (0.703–1.550)	0.833	269.505

OR – odds ratio; AIC – Akaike information criteria; CI – confidence interval; p-value – significance level; Bonferroni corrected significance level when *p* = 0.05/30; the bolded results indicate significant differences between the groups.

### 3.3. Distribution of *VEGFA* (rs1570360, rs699947, rs3025033, rs2146323, rs1413711, rs833061) genotypes and alleles among multiple sclerosis patients and controls by age

Age is an important factor influencing genetic associations [42]. Therefore, we stratified subjects using 38 years (the median age of our cohort) to evaluate *VEGFA* (rs1570360, rs699947, rs3025033, rs2146323, rs1413711 and rs833061) genotype and allele frequencies. No statistically significant differences were found between younger participants (≤38 years) in the multiple sclerosis and control groups (Table 8).

**Table 8.** Distribution of *VEGFA* rs1570360, rs699947, rs3025033, rs2146323, rs1413711 and rs833061 genotypes and alleles between younger (***< =***38 years) participants in multiple sclerosis patients and control groups.

Genotype/Allele	Control group	Multiple sclerosis	p-value
	n = 133	n = 143	
	n (%)	n (%)	
***< =38***			
***VEGFA rs1570360***			
GG	49 (36.8)	59 (41.3)	0.168
AG	56 (42.1)	66 (46.2)	** **
AA	28 (21.1)	18 (12.6)	
G	154 (57.9)	184 (64.3)	0.121
A	112 (42.1)	102 (35.7)	
***VEGFA rs699947***			
AA	44 (33.1)	45 (31.5)	0.708
AC	62 (46.6)	63 (44.1)	
CC	27 (20.3)	35 (24.5)	
A	150 (56.4)	153 (53.5)	0.438
C	116 (43.6)	133 (46.5)	
***VEGFA rs3025033***			
AA	83 (62.4)	90 (62.9)	0.111
AG	42 (31.6)	51 (35.7)	** **
GG	8 (6.0)	2 (1.4)	
A	208 (78.2)	231 (80.8)	0.454
G	58 (21.8)	55 (19.2)	
***VEGFA rs2146323***			
CC	51 (38.3)	67 (47.9)	0.339
AC	54 (40.6)	48 (33.6)	** **
AA	28 (21.1)	28 (19.6)	
C	156 (58.6)	104 (36.4)	0.230
A	110 (41.4)	182 (63.6)	
***VEGFA rs1413711***			
TT	48 (36.1)	44 (30.8)	0.628
CT	58 (43.6)	66 (46.2)	** **
CC	27 (20.3)	33 (23.1)	
T	154 (57.9)	154 (53.8)	0.339
C	112 (42.1)	132 (46.2)	
***VEGFA rs833061***			
CC	48 (36.1)	45 (31.5)	0.693
CT	58 (43.6)	66 (45.5)	** **
TT	27 (20.3)	33 (23.1)	
C	154 (57.9)	155 (54.2)	0.382
T	112 (42.1)	131 (45.8)	

p-value – significance level and Bonferroni corrected the significance level when *p* = 0.05/30; the bolded results indicate significant differences between the groups.

However, nominal differences in genotype distribution were observed for rs699947 (*p* = 0.030), rs1413711 (*p* = 0.011) and rs833061 (*p* = 0.025), with a higher frequency of variant genotypes (CC or TT) in MS patients compared to controls. Similarly, allele frequency analysis revealed nominal differences for rs699947 (*p* = 0.014), rs2146323 (*p* = 0.022), rs1413711 (*p* = 0.008) and rs833061 (*p* = 0.011), with minor alleles more frequently observed in the MS group. No statistically significant differences were identified for rs1570360 or rs3025033. However, after applying Bonferroni correction for multiple testing, none of the observed associations remained statistically significant and should therefore be interpreted with caution.

In contrast, analysis of older participants (>38 years) revealed statistically significant differences in genotype and allele distributions between MS patients and controls for several *VEGFA* variants (Table 9). However, after applying Bonferroni correction for multiple testing, these associations did not remain statistically significant.

**Table 9.** Distribution of *VEGFA* rs1570360, rs699947, rs3025033, rs2146323, rs1413711 and rs833061 genotypes and alleles between older participants (***>***38 years) in multiple sclerosis patients and control groups.

Genotype/Allele	Control group	Multiple sclerosis	p-value
	n = 137	n = 127	
	n (%)	n (%)	
***>38***			
***VEGFA rs1570360***			
GG	71 (51.8)	58 (45.7)	0.269
AG	40 (29.2)	49 (38.6)	** **
AA	26 (19.0)	20 (15.7)	
G	182 (66.4)	165 (65.0)	0.723
A	92 (33.6)	89 (35.0)	
***VEGFA rs699947***			
AA	41 (29.9)	21 (16.5)	0.030
AC	65 (47.4)	67 (52.8)	
CC	31 (22.6)	39 (30.7)	
A	147 (53.6)	109 (42.9)	0.014
C	127 (46.4)	145 (57.1)	
***VEGFA rs3025033***			
AA	96 (70.1)	77 (60.6)	0.168
AG	33 (24.1)	44 (34.6)	** **
GG	8 (5.8)	6 (4.7)	
A	225 (82.1)	198 (78.0)	0.231
G	49 (17.9)	56 (22.0)	
***VEGFA rs2146323***			
CC	58 (42.3)	72 (56.7)	0.061
AC	64 (46.7)	46 (36.2)	** **
AA	15 (10.9)	9 (7.1)	
C	180 (65.7)	190 (74.8)	0.022
A	94 (34.3)	64 (25.2)	
***VEGFA rs1413711***			
TT	43 (31.4)	20 (15.7)	0.011
CT	62 (45.3)	68 (53.5)	** **
CC	32 (23.4)	39 (30.7)	
T	148 (54.0)	108 (42.5)	0.008
C	126 (46.0)	146 (57.5)	
***VEGFA rs833061***			
CC	43 (31.4)	22 (17.3)	0.025
CT	62 (45.3)	65 (51.2)	** **
TT	32 (23.4)	40 (31.5)	
C	148 (54.0)	109 (42.9)	0.011
T	126 (46.0)	145 (57.1)	

p-value – significance level and Bonferroni corrected the significance level when *p* = 0.05/30; the bolded results indicate significant differences between the groups.

The binary logistic regression analysis showed no statistically significant associations between MS patients and controls among participants aged 38 years or younger (Table 10). In contrast, among participants older than 38 years, several nominal associations were observed (Table 11). In particular, rs699947 showed increased odds of MS under the co-dominant (AC vs. AA: OR = 2.012, *p* = 0.029; CC vs. AA: OR = 2.456, *p* = 0.013), dominant (OR = 2.156, *p* = 0.011) and additive models (OR = 1.548, *p* = 0.014). For rs1413711, increased odds of MS were observed under the co-dominant (CT vs. TT: OR = 2.358, *p* = 0.008; CC vs. TT: OR = 2.620, *p* = 0.008), dominant (OR = 2.447, *p* = 0.003) and additive models (OR = 1.587, *p* = 0.009). Similarly, rs833061 demonstrated nominal associations under the co-dominant (CT vs. CC: OR = 2.049, *p* = 0.023; TT vs. CC: OR = 2.443, *p* = 0.012), dominant (OR = 2.183, *p* = 0.009) and additive models (OR = 1.545, *p* = 0.013). In contrast, rs2146323 showed nominally decreased odds of MS under the co-dominant (AC vs. CC: OR = 0.579, *p* = 0.037), dominant (OR = 0.561, *p* = 0.020) and additive models (OR = 0.644, *p* = 0.024). No consistent associations were observed for rs1570360 or rs3025033. However, after applying Bonferroni correction for multiple testing, none of the observed associations remained statistically significant and should therefore be interpreted with caution.

**Table 10.** Binary logistic regression analysis of *VEGFA* rs1570360, rs699947, rs3025033, rs2146323, rs1413711 and rs833061 across younger participants (*< =*38 years) in multiple sclerosis patients and control groups.

Model	Genotype/allele	OR (95% CI)*	p-value	AIC
**< =38**				
***VEGFA rs1570360***				
Co-dominant	AG vs. GG	0.979 (0.582–1.646)	0.936	382.678
	AA vs. GG	0.534 (0.264–1.078)	0.080	
Dominant	AG+AA vs. GG	0.831 (0.511–1.349)	0.453	383.690
Recessive	AA vs. AG+GG	0.540 (0.283–1.031)	0.062	380.685
Overdominant	AG vs. AA+GG	1.179 (0.732–1.897)	0.499	383.797
Additive	A	0.775 (0.555–1.082)	0.134	381.997
***VEGFA rs699947***				
Co-dominant	AC vs. AA	0.994 (0.577–1.711)	0.981 0.476	385.563
	CC vs. AA	1.267 (0.661–2.432)		
Dominant	AC+CC vs. AA	1.077 (0.650–1.784)	0.774	384.173
Recessive	CC vs. AC+AA	1.272 (0.720–2.248)	0.407	383.563
Overdominant	AC vs. AA+CC	0.902 (0.561–1.449)	0.669	384.073
Additive	C	1.114 (0.807–1.538)	0.512	381.825
***VEGFA rs3025033***				
Co-dominant	AG vs. AA	1.120 (0.675–1.857)	0.661 0.068	381.607
	GG vs. AA	0.231 (0.048–1.117)		
Dominant	AG+GG vs. AA	0.927 (0.600–1.593)	0.927	384.247
Recessive	GG vs. AG+AA	0.222 (0.046–1.063)	0.060	379.799
Overdominant	AG vs. AA+GG	1.201 (0.728–1.982)	0.473	383.739
Additive	G	0.849 (0.557–1.294)	0.446	383.674
***VEGFA rs2146323***				
Co-dominant	AC vs. CC AA vs. CC	0.677 (0.397–1.153) 0.761 (0.402–1.441)	0.151 0.402	384.088
Dominant	AC+AA vs. CC	0.705 (0.437–1.140)	0.154	382.213
Recessive	AA vs. AC+CC	0.913 (0.508–1.442)	0.761	384.163
Overdominant	AC vs. AA+CC	0.739 (0.453–1.207)	0.227	382.791
Additive	A	0.842 (0.617–1.149)	0.277	383.071
***VEGFA rs1413711***				
Co-dominant	CT vs. TT	1.241 (0.723–2.131)	0.433	385.326
	CC vs. TT	1.333 (0.694–2.561)	0.388	
Dominant	CT+CC vs. TT	1.271 (0.770–2.098)	0.349	383.377
Recessive	CC vs. CT+TT	1.178 (0.663–2.092)	0.577	383.942
Overdominant	CT vs. CC+TT	1.108 (0.689–1.782)	0.671	384.074
Additive	C	1.163 (0.842–1.607)	0.360	383.413
***VEGFA rs833061***				
Co-dominant	CT vs. CC	1.195 (0.697–2.050)	0.516	385.521
	TT vs. CC	1.304 (0.680–2.500)	0.425	
Dominant	CT+TT vs. CC	1.230 (0.746–2.027)	0.417	383.596
Recessive	TT vs. CT+CC	1.178 (0.663–2.092)	0.577	383.942
Overdominant	CT vs. TT+CC	1.078 (0.670–1.733)	0.758	384.160
Additive	T	1.147 (0.831–1.583)	0.404	383.556

OR – odds ratio; AIC – Akaike information criteria; CI – confidence interval; p-value – significance level; Bonferroni corrected significance level when *p* = 0.05/30; the bolded results indicate significant differences between the groups.

**Table 11.** Binary logistic regression analysis of *VEGFA* rs1570360, rs699947, rs3025033, rs2146323, rs1413711 and rs833061 across older participants (***>***38 years) in multiple sclerosis patients and control groups.

Model	Genotype/allele	OR (95% CI)*	p-value	AIC
***>*38 years**				
***VEGFA rs1570360***				
Co-dominant	AG vs. GG	1.500 (0.871–2.581)	0.144	366.973
	AA vs. GG	0.942 (0.478–1.856)	0.862	
Dominant	AG+AA vs. GG	1.280 (0.789–2.076)	0.318	366.603
Recessive	AA vs. AG+GG	0.798 (0.421–1.421)	0.490	367.123
Overdominant	AG vs. AA+GG	1.523 (0.912–2.545)	0.108	365.003
Additive	A	1.053 (0.764–1.453)	0.752	367.503
***VEGFA rs699947***				
Co-dominant	AC vs. AA	2.012 (1.075–3.767)	0.029 0.013	362.467
	CC vs. AA	2.456 (1.212–4.978)		
Dominant	AC+CC vs. AA	2.156 (1.190–3.905)	0.011	360.918
Recessive	CC vs. AC+AA	1.515 (0.874–2.626)	0.138	365.393
Overdominant	AC vs. AA+CC	1.237 (0.763–2.006)	0.389	366.859
Additive	C	1.548 (1.091–2.197)	0.014	361.465
***VEGFA rs3025033***				
Co-dominant	AG vs. AA	1.662 (0.967–2.858)	0.066 0.905	366.027
	GG vs. AA	0.935 (0.311–2.810)		
Dominant	AG+GG vs. AA	1.520 (0.913–2.533)	0.108	364.999
Recessive	GG vs. AG+AA	0.800 (0.270–2.371)	0.687	367.439
Overdominant	AG vs. AA+GG	1.671 (0.978–2.854)	0.060	364.042
Additive	G	1.273 (0.843–1.924)	0.251	366.279
***VEGFA rs2146323***				
Co-dominant	AC vs. CC AA vs. CC	0.579 (0.347–0.967) 0.483 (0.197–1.184)	0.037 0.112	363.996
Dominant	AC+AA vs. CC	0.561 (0.344–0.914)	0.020	362.149
Recessive	AA vs. AC+CC	0.620 (0.261–1.472)	0.279	366.399
Overdominant	AC vs. AA+CC	0.648 (0.395–1.061)	0.085	364.607
Additive	A	0.644 (0.440–0.943)	0.024	362.367
***VEGFA rs1413711***				
Co-dominant	CT vs. TT	2.358 (1.253–4.438)	**0.008**	360.419
	CC vs. TT	2.620 (1.292–5.314)	**0.008**	
Dominant	CT+CC vs. TT	2.447 (1.345–4.453)	**0.003**	358.546
Recessive	CC vs. CT+TT	1.454 (0.842–2.512)	0.179	365.791
Overdominant	CT vs. CC+TT	1.394 (0.859–2.263)	0.179	365.790
Additive	C	1.587 (1.120–2.249)	0.009	360.666
***VEGFA rs833061***				
Co-dominant	CT vs. CC	2.049 (1.102–3.811)	0.023	362.112
	TT vs. CC	2.443 (1.222–4.885)	0.012	
Dominant	CT+TT vs. CC	2.183 (1.217–3.916)	0.009	360.465
Recessive	TT vs. CT+CC	1.509 (0.875–2.601)	0.139	365.401
Overdominant	CT vs. TT+CC	1.268 (0.782–2.058)	0.336	366.675
Additive	T	1.545 (1.096–2.179)	0.013	361.284

OR – odds ratio; AIC – Akaike information criteria; CI – confidence interval; p-value – significance level; Bonferroni corrected significance level when *p* = 0.05/30; the bolded results indicate significant differences between the groups.

### 3.4. Comparison of serum VEGFA concentrations between multiple sclerosis patients and controls

Serum VEGFA levels were assessed in 40 MS patients and 40 healthy controls. No significant differences in age or sex distribution were observed between these groups (Table 1).

**Figure 1.**
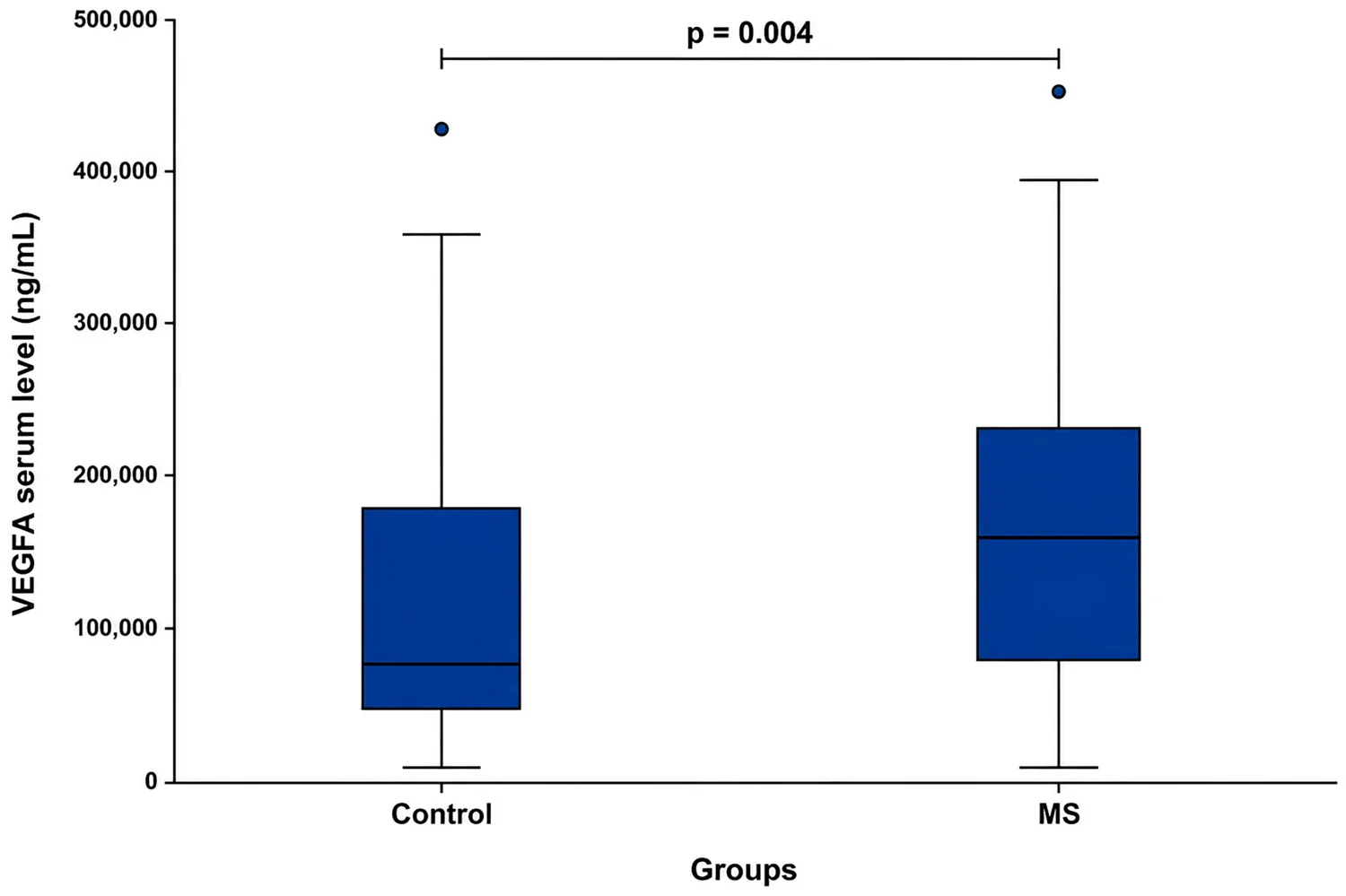
VEGFA serum levels in patients with multiple sclerosis and control group subjects. Mann-Whitney U test was used to assess serum VEGFA levels differences between patients with multiple sclerosis and control groups; *p* = 0.004.

The serum protein analysis results showed a significant difference in VEGFA serum concentrations between the two groups, with median levels of 151.864 pg/ml (IQR: 150.906) in MS patients and 66.695 pg/ml (IQR: 142.535) in controls (*p* = 0.004) (Figure 1).

Our study additionally examined the distribution of genotypes in relation to serum VEGFA levels and VEGFA polymorphisms rs1570360, rs699947, rs3025033, rs2146323 and rs833061, considering statistical significance at *p* < 0.05. Among the variants evaluated, MS patients with the rs1570360 AG genotype showed significantly elevated VEGFA serum concentrations compared to controls (149.837 [117.168] pg/ml vs. 56.978 [112.461] pg/ml, *p* = 0.004). Similarly, higher VEGFA levels were observed in MS patients carrying the rs699947 AA genotype (147.045 [145.599] pg/ml vs. 61.069 [116.175] pg/ml, *p* = 0.039), rs3025033 AA genotype (117.559 [151.755] pg/ml vs. 65.588 [69.698] pg/ml, *p* = 0.036), rs2146323 AC genotype (178.720 [105.854] pg/ml vs. 52.186 [2.763] pg/ml, *p* = 0.010) and rs833061 AC genotype (139.606 [134.019] pg/ml vs. 61.069 [116.175] pg/ml, *p* = 0.044) (Table 12).

**Table 12.** Genotype distribution and serum VEGFA levels between patients with multiple sclerosis and control group.

Genotype	VEGFA level (pg/mL)		P-value
	Control Median (IQR)	Multiple Sclerosis Median (IQR)	
***VEGFA rs1570360***			
GG	61.069 (69.992)	163.633 (221.540)	0.051
AG	56.978 (112.461)	149.837 (117.168)	**0.004**
AA	186.431 (241.287)	129.722 (110.467)	0.372
***VEGFA rs699947***			
AA	61.069 (116.175)	147.045 (145.599)	**0.039**
AC	93.422 (156.169)	152.675 (135.554)	0.146
CC	55.137 (41.476)	163.633 (124.123)	0.138
***VEGFA rs3025033***			
AA	65.588 (69.698)	117.559 (151.755)	**0.036**
AG	74.734 (244.588)	179.470 (115.389)	0.155
GG	62.092 (283.374)	–	0.157
***VEGFA rs2146323***			
CC	59.210 (76.781)	76.321 (123.839)	0.378
AC	52.186 (2.763)	178.720 (105.854)	**0.010**
AA	90.497 (163.416)	202.919 (244.637)	0.203
***VEGFA rs1413711***			
TT	61.069 (116.175)	145.468 (133.494)	0.052
CT	93.422 (156.169)	173.498 (123.469)	0.114
CC	55.137 (41.476)	163.633 (186.014)	0.138
***VEGFA rs833061***			
CC	61.069 (116.175)	139.606 (134.019)	**0.044**
CT	93.422 (156.169)	194.322 (135.554)	0.113
TT	55.137 (41.476)	159.649 (186.014)	0.138

* Student T-test; p-value – significance level when *p* = 0.05; the bolded results indicate significant differences between the groups.

### 3.5. *VEGFA* haplotype analysis

Pairwise linkage disequilibrium (LD) analysis was performed among the *VEGFA* polymorphisms in patients with multiple sclerosis (Table 13). Strong LD was observed between several SNP pairs, particularly rs699947–rs1413711 (D′ = 0.985, r^2^ = 0.952), rs699947–rs833061 (D′ = 0.989, r^2^ = 0.952) and rs1413711–rs833061 (D′ = 0.993, r^2^ = 0.978). High LD was also identified between rs699947 and rs2146323 (D′ = 0.974, r^2^ = 0.464), as well as between rs2146323 and both rs1413711 (D′ = 0.994, r^2^ = 0.474) and rs833061 (D′ = 0.993, r^2^ = 0.471). Moderate LD was observed between rs1570360 and rs699947, rs1413711 and rs833061 (D′ = 0.731–0.754; r^2^ = 0.287–0.299). In contrast, rs3025033 exhibited only weak LD with most variants and no evidence of LD with rs2146323 (D′ = 0.039, r^2^ = 0.001, *p* = 0.376). Overall, these findings demonstrate that most VEGFA polymorphisms analyzed are inherited in strong linkage disequilibrium, supporting the observed haplotype structure within the *VEGFA* locus.

**Table 13.** Linkage disequilibrium between every two *VEGFA* SNPs.

SNP-SNP	D’	r2	p-value
rs1570360–rs699947	0.7311	0.287	0
rs1570360–rs3025033	0.2517	0.009	**0.0016**
rs1570360–rs2146323	0.1444	0.019	0
rs1570360–*rs1413711*	0.7539	0.299	0
rs1570360–rs833061	0.7467	0.292	0
rs699947-*rs3025033*	0.2548	0.018	0
rs699947-*rs2146323*	0.9735	0.464	0
rs699947-rs1413711	0.985	0.952	0
rs699947-rs833061	0.9886	0.952	0
rs3025033-rs2146323	0.0388	0.001	0.3763
rs3025033-rs1413711	0.2073	0.012	**0.0003**
rs3025033-rs833061	0.2127	0.013	**0.0002**
rs2146323–rs1413711	0.9935	0.474	0
rs2146323–rs833061	0.9932	0.471	0
rs1413711–rs833061	0.9925	0.978	0

SNP – single nucleotide polymorphism; D’: the deviation between the expected haplotype frequency and the observed frequency; r^2^: the square of the haplotype frequency correlation coefficient; p-value – significance level when *p* < 0.05.

Haplotype analysis of *VEGFA* polymorphisms (rs1570360, rs699947, rs3025033, rs2146323, rs1413711 and rs833061) was performed in patients with MS and presented in Table 14. Two haplotypes (A-A-A-A-T-C and G-A-G-A-T-C) showed nominal associations with reduced odds of MS (OR = 0.62, 95% CI: 0.380–0.990, *p* = 0.046; and OR = 0.37, 95% CI: 0.140–0.940, *p* = 0.037, respectively). However, these associations did not remain significant after correction for multiple testing and should be interpreted with caution. The observed haplotype structure is consistent with the strong linkage disequilibrium between several *VEGFA* variants.

**Table 14.** Haplotype association of *VEGFA* rs1570360, rs699947, rs3025033, rs2146323, rs1413711 and rs833061 with the predisposition to MS occurrence.

Haplotype No.	*VEGFA rs1570360*	*VEGFA rs699947*	*VEGFA rs3025033*	*VEGFA rs2146323*	*VEGFA rs1413711*	*VEGFA rs833061*	Frequency		OR (95% CI)	p-value
							MS	Reference		
1	G	C	A	C	C	T	33.16	31.86	1	NA
2	A	A	A	C	T	C	16.65	17.04	0.890 (0.610–1.290)	0.540
3	G	A	A	A	T	C	16.6	15.31	1.080 (0.730–1.590)	0.700
4	G	C	G	C	C	T	12.03	9.04	1.340 (0.820–2.210)	0.240
5	A	A	A	A	T	C	8.09	12.2	0.620 (0.380–0.990)	**0.046**
6	A	A	G	A	T	C	4.14	4.43	0.980 (0.470–2.040)	0.960
7	A	C	A	C	C	T	4.19	2.52	2.130 (0.95–4.77)	0.066
8	G	A	G	A	T	C	2.228	4.73	0.370 (0.14–0.94)	**0.037**

OR: odds ratio; CI: confidence interval; p-value: significance level (statistically significant when *p* < 0.05/7).

### 3.6. Clinical characteristics of multiple slcerosis and their association with VEGFA SNP genotypes

The median (IQR) duration of MS in the MS group was 5 (7), with a median age at diagnosis of 30 (14). The EDSS score was 2.5 (3.0). The disease phenotypes within the MS group were distributed as follows: RRMS + CIS (46.7%) and PPMS + SPMS (53.1%). Regarding symptom presence, 43.3% of patients presented with pyramidal symptoms, 30.2% had sensory disturbances, 25.9% showed brainstem involvement, 28.0% exhibited cerebellar symptoms, 17.2% had pelvic organ dysfunction and 27.4% reported visual symptoms. Disease phenotypes and affected functional systems in the MS group are presented in the Table 15.

**Table 15.** Multiple sclerosis group disease phenotypes and affected functional systems.

Variable	Gender		p value
	Women n (%)	Men n (%)	
**RRMS + CIS**	162 (46.7)	90 (46.9)	0.966
**PPMS + SPMS**	185 (53.3)	102 (53.1)	
Pyramidal system symptoms n (%)	148 (85.5)	86 (89.6)	0.346
Sensory system symptoms n (%)	113 (65.3)	50 (52.1)	0.033
Brainstem system symptoms n (%)	93 (53.8)	47 (49.0)	0.450
Cerebellar system symptoms n (%)	98 (56.6)	53 (55.2)	0.820
Pelvic system symptoms n (%)	62 (35.8)	31 (32.3)	0.558
Visual system symptoms n (%)	94 (54.3)	54 (56.3)	0.762

CIS – clinically isolated syndrome, RR – relapsing-remitting, SP – secondary progressive, PP – primary progressive.

Correlation analysis was performed to explore relationships between clinical variables, VEGFA serum levels and *VEGFA* polymorphisms. As expected, age showed a strong positive correlation with age at MS diagnosis (*r* = 0.854, *p* < 0.001) and moderate correlations with disease duration (*r* = 0.456, *p* < 0.001) and EDSS (*r* = 0.337, *p* < 0.001). Disease duration was also positively correlated with EDSS (*r* = 0.369, *p* < 0.001), reflecting known clinical dependencies. A weak correlation was observed between age at MS diagnosis and rs2146323 (r = −0.122, *p* = 0.046); however, this finding should be interpreted with caution. Strong correlations were identified among several *VEGFA* polymorphisms, particularly between rs699947, rs1413711 and rs833061 (*r* = 0.977–0.989, *p* < 0.001), consistent with linkage disequilibrium within the *VEGFA* locus. No significant correlations were observed between VEGFA serum levels and clinical or genetic variables. Statistically significant correlations are presented in Table 16.

**Table 16.** Multiple sclerosis group quantitative clinical characteristic, *VEGFA* serum concentration and *VEGFA* (rs1570360, rs699947, rs3025033, rs2146323, rs1413711 and rs833061) allele count Correlation matrix.

Variable 1	Variable 2	r	p value
Age	Age at MS diagnosis	0.854	<0.001
Age	MS duration (years)	0.456	<0.001
Age	EDSS	0.337	<0.001
Age at MS diagnosis	EDSS	0.203	<0.001
MS duration (years)	EDSS	0.369	<0.001
Age at MS diagnosis	rs2146323	−0.122	0.046
rs1570360	rs699947	−0.598	<0.001
rs1570360	rs3025033	−0.086	0.047
rs1570360	rs2146323	0.152	<0.001
rs1570360	rs1413711	−0.610	<0.001
rs1570360	rs833061	−0.606	<0.001
rs699947	rs3025033	0.138	0.001
rs699947	rs2146323	−0.685	<0.001
rs699947	rs1413711	0.977	<0.001
rs699947	rs833061	0.977	<0.001
rs3025033	rs1413711	0.112	0.009
rs3025033	rs833061	0.115	0.007
rs2146323	rs1413711	−0.696	<0.001
rs2146323	rs833061	−0.686	<0.001
rs1413711	rs833061	0.989	<0.001

Spearman correlation coefficient (r) and statistically significant p value are shown in the correlation matrix MS – multiple sclerosis, EDSS – expanded disability status scale.

No statistically significant differences in *VEGFA* (rs1570360, rs699947, rs3025033, rs2146323, rs1413711 and rs833061) genotype distribution were observed between RRMS + CIS and PPMS + SPMS phenotype subgroups (Table 17).

**Table 17.** Analysis of multiple sclerosis phenotype and *VEGFA* (rs1570360, rs699947, rs3025033, rs2146323, rs1413711 and rs833061) genotype distribution.

*VEGFA* SNP		Phenotype – n (%)		
	Genotype	RRMS + CIS	PPMS + SPMS	P value
rs1570360	GG	112 (44.4)	124 (43.2)	0.056
	AG	107 (42.5)	104 (36.2)	
	AA	33 (13.1)	59 (20.6)	
rs699947	AA	60 (23.8)	91 (31.7)	0.068
	AC	122 (48.4)	135 (47.0)	
	CC	70 (27.8)	61 (21.3)	
rs3025033	AA	152 (60.3)	193 (67.2)	0.040
	AG	92 (36.5)	78 (27.2)	
	GG	8 (3.2)	16 (5.6)	
rs2146323	CC	128 (50.8)	119 (41.5)	0.083
	AC	88 (34.9)	124 (43.2)	
	AA	36 (14.3)	44 (15.3)	
rs1413711	TT	59 (23.4)	96 (33.4)	0.031
	CT	125 (49.6)	129 (44.9)	
	CC	68 (27.0)	62 (21.6)	
rs833061	CC	60 (23.8)	98 (34.1)	0.026
	CT	123 (48.8)	127 (44.3)	
	TT	69 (27.4)	62 (21.6)	

SNP – single nucleotide polymorphism, CIS – clinically isolated syndrome, RR – relapsing-remitting, SP – secondary progressive, PP – primary progressive. Bonferroni corrected significance level *p* < 0.0083.

We analyzed whether associations existed between the prevalence of affected functional systems and *VEGFA* (rs1570360, rs699947, rs3025033, rs2146323, rs1413711 and rs833061) genotypes. We found that cerebellar involvement was more frequent in patients with the rs1570360 AG genotype compared with other genotypes (*p* = 0.0008). In addition, pelvic symptoms were less frequent in the rs2146323 AC genotype compared with other genotypes (*p* = 0.002). The results are shown in Table 18.

**Table 18.** Analysis of affected functional systems and *VEGFA* (rs1570360, rs699947, rs3025033, rs2146323, rs1413711 and rs833061) genotype distribution.

*VEGFA* SNP	Genotype		Patients affected by symptoms in different systems n (%)					
			Pyramidal	Sensory	Brainstem	Cerebellar	Pelvic	Visual
*rs1570360*	GG		101 (43.2)	68 (41.7)	58 (41.4)	57 (37.7)	43 (46.2)	70 (47.3)
	AG		99 (42.3)	74 (45.4)	67 (47.9)	78 (51.7)	38 (40.9)	62 (41.9)
	AA		34 (14.5)	21 (12.9)	15 (10.7)	16 (10.6)	12 (12.9)	16 (10.8)
p value			0.865	0.517	0.112	**0.003**	0.745	0.139
*rs699947*	AA		53 (22.6)	41 (25.2)	36 (25.7)	39 (25.8)	20 (21.5)	36 (24.3)
	AC		117 (50.0)	83 (50.9)	69 (49.3)	78 (51.7)	51 (54.8)	70 (47.3)
	CC		64 (27.4)	39 (23.9)	35 (25.0)	34 (22.5)	22 (23.7)	42 (28.4)
p value			0.159	0.331	0.700	0.155	0.299	0.876
*rs3025033*	AA		149 (63.7)	98 (60.1)	86 (61.4)	96 (63.6)	58 (62.4)	95 (64.2)
	AG		77 (32.9)	59 (36.2)	49 (35.0)	50 (33.1)	31 (33.3)	47 (31.8)
	GG		8 (3.4)	6 (3.7)	5 (3.6)	5 (3.3)	4 (4.3)	6 (4.1)
p value			0.071	0.620	0.834	0.671	0.604	0.247
*rs2146323*	CC		121 (51.7)	79 (48.5)	67 (47.9)	72 (47.7)	38 (40.9)	23 (15.5)
	AC		84 (35.9)	60 (36.8)	51 (36.4)	58 (38.4)	44 (47.3)	53 (35.8)
	AA		29 (12.4)	24 (14.7)	22 (15.7)	21 (13.9)	11 (22.8)	72 (48.6)
p value			0.229	0.511	0.433	0.356	**0.008**	0.526
*rs1413711*	TT		51 (21.8)	39 (23.9)	35 (25.0)	37 (24.5)	19 (20.4)	34 (23.0)
	CT		121 (51.7)	87 (53.4)	71 (50.7)	82 (54.3)	52 (55.9)	73 (49.3)
	CC		62 (26.5)	37 (22.7)	34 (24.3)	32 (21.2)	22 (23.7)	41 (27.7)
p value			0.115	0.202	0.698	0.082	0.343	0.851
*rs833061*	CC		54 (23.1)	42 (25.8)	38 (27.1)	40 (26.5)	22 (23.7)	37 (25.0)
	CT		117 (50.0)	84 (51.5)	67 (47.9)	78 (51.7)	49 (52.7)	70 (47.3)
	TT		9 (25.7)	37 (22.7)	35 (25.0)	33 (21.9)	22 (23.7)	41 (27.7)
p value			0.176	0.169	0.625	0.120	0.559	0.913

SNP – single nucleotide polymorphism. Bonferroni corrected significance level *p* < 0.0083.

As symptom prevalence was not evenly distributed between genders, we analyzed the prevalence of affected functional systems after stratifying subjects by sex. We found that pyramidal symptoms were more frequent in women carrying the rs699947 C allele (AC + CC vs. AA, *p* = 0.002; Table 19). Additionally, in men, cerebellar symptoms were more common in those with the rs1570360 AG genotype compared with other genotypes (*p* = 0.002), while sensory symptoms were less frequent in individuals with the rs833061 TT genotype compared with other genotypes (*p* = 0.004). The results of genotype distribution in men are presented in Table 20.

**Table 19.** Gender-stratified analysis of affected functional systems and *VEGFA* (rs1570360, rs699947, rs3025033, rs2146323, rs1413711 and rs833061) genotype distribution between women.

Women								
*VEGFA* SNP	Genotype		Patients affected by symptoms in different systems n (%)					
			Pyramidal	Sensory	Brainstem	Cerebellar	Pelvic	Visual
*rs1570360*	GG		18 (12.2)	54 (47.8)	42 (45.2)	43 (43.9)	32 (51.6)	52 (55.3)
	AG		60 (40.5)	47 (41.6)	42 (45.2)	46 (46.9)	22 (35.5)	35 (37.2)
	AA		70 (47.3)	12 (10.6)	9 (9.7)	9 (9.2)	8 (12.9)	7 (7.4)
p value			0.825	0.867	0.436	0.164	0.538	0.039
*rs699947*	AA		29 (19.6)	24 (21.2)	21 (22.6)	22 (22.4)	9 (14.5)	25 (26.6)
	AC		70 (47.3)	55 (48.7)	43 (46.2)	48 (49.0)	33 (53.2)	35 (37.2)
	CC		49 (33.1)	34 (30.1)	29 (31.2)	28 (28.6)	20 (32.3)	34 (36.2)
p value			**0.006**	0.220	0.804	0.291	0.069	0.153
*rs3025033*	AA		87 (58.8)	68 (60.2)	55 (59.1)	61 (62.2)	37 (59.7)	55 (58.5)
	AG		54 (36.5)	39 (34.5)	34 (36.6)	33 (33.7)	21 (33.9)	33 (35.1)
	GG		7 (4.7)	6 (5.3)	4 (4.3)	2 (4.1)	4 (6.5)	6 (6.4)
p value			0.351	0.255	0.892	0.375	0.386	0.192
*rs2146323*	CC		75 (50.7)	56 (49.6)	45 (48.4)	46 (46.9)	26 (41.9)	42 (44.7)
	AC		54 (36.5)	40 (35.4)	33 (35.5)	38 (38.8)	30 (48.4)	36 (38.3)
	AA		19 (12.8)	17 (15.0)	15 (16.1)	14 (14.3)	6 (9.7)	16 (17.0)
p value			0.051	0.961	0.970	0.531	0.019	0.442
*rs1413711*	TT		30 (20.3)	25 (22.1)	22 (23.7)	23 (23.5)	10 (16.1)	25 (26.6)
	CT		69 (46.6)	54 (47.8)	42 (45.2)	67 (48.0)	32 (51.6)	35 (37.2)
	CC		49 (33.1)	34 (30.1)	29 (31.2)	28 (28.6)	20 (32.3)	34 (36.2)
p value			0.009	0.269	0.873	0.337	0.125	0.207
*rs833061*	CC		30 (20.3)	25 (22.1)	22 (23.7)	23 (23.5)	10 (16.1)	25 (26.6)
	CT		69 (46.6)	54 (47.8)	42 (45.2)	47 (48.0)	32 (51.6)	35 (37.2)
	TT		49 (33.1)	34 (30.1)	29 (31.2)	28 (28.6)	20 (32.3)	34 (36.2)
p value			0.009	0.269	0.873	0.337	0.125	0.207

SNP – single nucleotide polymorphism. Bonferroni corrected significance level *p* < 0.0083.

**Table 20.** Gender-stratified analysis of affected functional systems and *VEGFA* (rs1570360, rs699947, rs3025033, rs2146323, rs1413711 and rs833061) genotype distribution between men.

Men								
*VEGFA* SNP	Genotype		Patients affected by symptoms in different systems n (%)					
			Pyramidal	Sensory	Brainstem	Cerebellar	Pelvic	Visual
*rs1570360*	GG		31 (36.0)	14 (28.0)	16 (34.0)	14 (26.4)	11 (35.5)	18 (33.3)
	AG		39 (45.3)	27 (54.0)	25 (53.2)	32 (60.4)	16 (51.6)	27 (50.0)
	AA		16 (18.6)	9 (18.0)	6 (12.8)	7 (13.2)	4 (12.9)	9 (16.7)
p value			0.931	0.205	0.235	**0.006**	0.558	0.636
*rs699947*	AA		24 (27.9)	17 (34.0)	15 (31.9)	17 (32.1)	11 (35.5)	11 (20.4)
	AC		47 (54.7)	28 (56.0)	26 (55.3)	30 (56.6)	18 (58.1)	35 (64.8)
	CC		15 (17.4)	5 (10.0)	6 (12.8)	6 (11.3)	2 (6.5)	8 (14.8)
p value			0.470	0.049	0.286	0.112	0.088	0.156
*rs3025033*	AA		62 (72.1)	30 (60.0)	31 (66.0)	35 (66.0)	21 (67.7)	40 (74.1)
	AG		23 (26.7)	20 (40.0)	15 (31.9)	17 (32.1)	10 (32.3)	14 (25.9)
	GG		1 (1.2)	0 (0.0)	1 (2.1)	1 (1.9)	0 (0.0)	0 (0.0)
p value			0.094	0.062	0.539	0.584	0.761	0.280
*rs2146323*	CC		46 (53.5)	23 (46.0)	22 (46.8)	26 (49.1)	12 (38.7)	30 (55.6)
	AC		30 (34.9)	20 (40.0)	18 (38.3)	20 (37.7)	14 (45.2)	17 (31.5)
	AA		10 (11.6)	7 (14.0)	7 (14.9)	7 (13.2)	5 (16.1)	7 (13.0)
p value			0.434	0.146	0.186	0.353	0.073	0.585
*rs1413711*	TT		21 (24.4)	14 (28.0)	13 (27.7)	14 (26.4)	9 (29.0)	9 (16.7)
	CT		52 (60.5)	33 (66.0)	29 (61.7)	35 (66.0)	20 (64.5)	38 (70.4)
	CC		13 (15.1)	3 (6.0)	5 (10.6)	4 (7.5)	2 (6.5)	7 (13.0)
p value			0.582	0.021	0.306	0.050	0.194	0.119
*rs833061*	CC		24 (27.9)	17 (34.0)	16 (34.0)	17 (31.1)	12 (38.7)	12 (22.2)
	CT		48 (55.8)	30 (60.0)	25 (53.2)	31 (58.5)	17 (54.8)	35 (64.8)
	TT		14 (16.3)	3 (6.0)	6 (12.8)	5 (9.4)	2 (6.5)	7 (13.0)
p value			0.474	**0.007**	0.185	0.067	0.057	0.231

SNP – single nucleotide polymorphism. Bonferroni corrected significance level *p* < 0.0083.

Finally, we examined the associations between VEGFA (rs1570360, rs699947, rs3025033, rs2146323, rs1413711 and rs833061) genotypes and clinical parameters, including age at MS diagnosis, disease duration and EDSS. No statistically significant associations were identified (Table 21).

**Table 21.** Analysis of disease characteristics and *VEGFA* (rs1570360, rs699947, rs3025033, rs2146323, rs1413711 and rs833061) genotype distribution.

*VEGFA* SNP	Genotype		Patients affected by symptoms in different systems n (%)		
			Age of MS diagnosis median (IQR)	MS duration years median (IQR)	EDSS median (IQR)
*rs1570360*	GG		31.2 (15.75)	7 (7)	25 (41)
	AG		30 (14.0)	5 (7)	25 (29)
	AA		29.5 (13)	6.5 (7.0)	15 (29)
p value			0.945	0.529	0.467
*rs699947*	AA		27 (13)	4.5 (8)	25 (29)
	AC		31 (14.25)	7 (7)	25 (32.5)
	CC		32 (14.5)	5 (8)	25 (41.5)
p value			0.023	0.063	0.873
*rs3025033*	AA		30 (14)	6.5 (7)	25 (29)
	AG		31 (16)	4 (8)	25 (40)
	GG		32 (15.5)	12 (6.25)	25 (46.75)
p value			0.999	0.009	0.535
*rs2146323*	CC		32 (57)	5 (7.25)	25 (31.75)
	AC		28 (15.25)	8 (7)	25 (40)
	AA		28 (10.5)	4 (6)	25 (24.5)
p value			0.128	0.085	0.849
*rs1413711*	TT		27 (12)	5 (8)	25 (28.88)
	CT		31 (14.25)	6.5 (7)	25 (39.25)
	CC		32 (15)	5 (8)	25 (42)
p value			0.013	0.233	0.693
*rs833061*	CC		28 (13)	5 (8)	25 (29)
	CT		30.5 (14)	7 (7)	25 (32.5)
	TT		32 (15)	5.5 (8)	25 (18.7)
p value			0.045	0.143	0.861

SNP – single nucleotide polymorphism, MS – multiple sclerosis, EDSS – expanded disability status scale, MSSS – multiple sclerosis severity score, IQR – interquartile range. Bonferroni corrected significance level *p* < 0.0083. Statistically significant results are shown in bold.

## 4. Discussion

In this study, we aimed to explore the relationship between *VEGFA* gene polymorphisms, VEGFA serum levels and their potential role in the development of MS in Lithuania. The findings showed that two of these gene variants were to increased susceptibility to MS. Additionally, five out of the six SNPs were significantly related to differences in VEGFA levels when comparing MS patients to controls. For some of these variants (rs1570360, rs699947, rs3025033, rs2146323 and rs833061), certain genotypes were linked to increased VEGFA levels in MS patients.

Although our analysis showed no significant differences in overall *VEGFA* genotype and allele frequencies between MS patients and healthy controls, our results highlight sex-specific genetic variations, with certain alleles being less frequent in female MS patients and associations between genotypes and the likelihood of MS differing between men and women. These findings suggest a potential gender-specific association of these *VEGFA* variants with MS susceptibility in women. Being female is one of the strongest factors associated with MS. The majority of MS patients are women, with a current sex ratio of approximately 3:1, and this ratio is increasing over time [43]. Female susceptibility to MS is influenced by sex-specific epigenetic changes, significant hormonal shifts during puberty and environmental factors compared to males, such as less sun exposure [44,45]. Genetically, one explanation involves differences in how genes on the X chromosome are regulated between men and women. Some X chromosome genes are expressed differently in female immune cells compared to males, which can affect inflammation [46]. Additionally, certain genes on the X chromosome that escape full inactivation in females may increase immune activity [47]. Beyond genetics, hormonal factors also play a significant role. Puberty, pregnancy and menopause (periods marked by dramatic changes in sex hormone levels) have been extensively studied. MS incidence is similar between boys and girls before puberty but rises sharply in girls after puberty, making puberty a significant risk factor [48]. Some studies report that MS can affect female reproductive health and pregnancy outcomes [49,50]. Environmental factors likely play a major role in MS risk, as indicated by a 75% discordance rate among identical twins [51]. In addition to findings in females, our study also identified a genotype in males (rs1413711 CT) that appears to be linked with an increased likelihood of developing MS, further supporting the role of sex-specific genetic influences in the disease. Notably, men with MS generally experience more aggressive disease courses and accumulate disability more quickly than [52].

Our age-based analysis revealed that the *VEGFA* rs1413711 CT+CC genotypes were associated with increased odds of MS in participants older than 38 years, while no significant associations were observed in younger individuals. This finding correlates with the well-established impact of aging on immune function and CNS pathology. As individuals age, their brains undergo structural and functional changes, including reduced plasticity, accumulation of intra- and extracellular proteins, loss of grey matter volume and increased white matter abnormalities [53]. MS contributes additional damage through inflammatory processes, demyelination and neurodegeneration [54]. In MS patients, the combined effects of aging and disease pathology interact in complex ways, potentially worsening brain damage beyond what occurs in either condition alone. Since the brain’s capacity for repair reduces with age, MS – related damage may have a stronger negative impact in older patients, leading to faster disease progression and more severe disability compared to younger patients with similar disease duration [55].

From an immunopathological perspective, aging induces changes in the CNS immune environment. The aging immune system becomes less effective and is characterized by chronic low-level inflammation, a phenomenon known as “inflammaging” [56]. These alterations affect key immune cells in both the brain and peripheral tissues, impairing the regulation of inflammation and tissue repair [57] In the context of MS, such immune changes may worsen disease level of impact and modulate the influence of genetic factors like *VEGFA* on disease susceptibility.

Our study used 38 years as the median age threshold for stratification, which matches with the typical age range studied in MS research. However, no specific clinical or epidemiological threshold at exactly 38 years has been noted in the literature. Previous studies have demonstrated that MS clinical manifestations vary by age at onset: younger patients typically experience a relapsing-remitting course, whereas older patients often exhibit more rapid progression to sustained [58]. Overall, our results highlight that age is a critical factor in MS pathogenesis and should be carefully considered in future genetic and clinical investigations.

Our findings also demonstrate that serum VEGFA levels differ significantly between MS patients and healthy individuals, suggesting a potential role of VEGFA alteration in MS pathogenesis. The observed nominal associations between VEGFA polymorphisms and circulating VEGFA concentrations suggest that genetic variation within the VEGFA locus may influence VEGFA expression; however, these findings should be interpreted cautiously. VEGFA is known to regulate angiogenesis and vascular permeability, processes that are critically involved in the inflammatory environment of MS lesions [28,32]. Altered VEGFA levels may therefore affect blood-brain barrier integrity and promote neuroinflammation, worsening disease progression. In the present study, nominally significant differences in serum VEGFA concentrations were observed across several VEGFA genotypes. These findings suggest that genetic variation within the VEGFA locus may contribute to interindividual variability in circulating VEGFA levels. However, given the exploratory nature of these analyses, the relatively small serum subgroup and the lack of comprehensive treatment data, these observations should be interpreted with caution and require validation in larger, well-characterized cohorts.

Pairwise linkage disequilibrium analysis demonstrated strong LD among several *VEGFA* polymorphisms, particularly between rs699947, rs1413711 and rs833061, as well as between rs699947 and rs2146323 and between rs2146323 and both rs1413711 and rs833061. These findings indicate that these variants are frequently inherited together and reflect the underlying genetic architecture of the *VEGFA* locus. Consistent with this LD structure, haplotype analysis identified two haplotypes that were nominally associated with reduced odds of MS. However, these associations did not remain statistically significant after correction for multiple testing and should therefore be interpreted with caution. Future studies in larger independent cohorts are required to determine whether specific VEGFA haplotypes contribute to MS susceptibility beyond the effects of individual variants.

The clinical presentation of multiple sclerosis is highly heterogeneous, reflecting complex interactions between inflammatory, neurodegenerative and vascular mechanisms within the central nervous system [2]. In the present study, evaluation of affected functional systems revealed genotype-dependent differences in symptom prevalence, suggesting that VEGFA polymorphisms may contribute to variability in clinical manifestations rather than to global measures of disease severity. Specifically, certain *VEGFA* variants were associated with differences in the frequency of cerebellar, pyramidal, sensory and pelvic symptoms, with several associations showing sex-specific patterns. These findings indicate that *VEGFA*-related pathways may influence regional vulnerability or symptom expression in MS, potentially through effects on angiogenesis, blood–brain barrier integrity or neuroinflammatory processes [19,28]. Notably, no associations were observed between *VEGFA* genotypes and disease duration or disability as measured by EDSS, supporting the notion that these genetic variants may modulate clinical phenotype without substantially affecting overall disease progression. While the biological mechanisms underlying these associations remain to be fully elucidated, the observed symptom-specific patterns highlight the importance of considering functional system involvement and sex stratification when exploring genetic contributions to MS heterogeneity.

Although most of the *VEGFA* gene variants analyzed here (rs1570360, rs699947, rs3025033, rs2146323, rs1413711 and rs833061) have not been commonly associated with MS in previous studies, their potential roles in regulating VEGFA expression and vascular or inflammatory pathways warrant investigation in the context of MS susceptibility.

Starting with rs1570360, this polymorphism is linked to autoimmune and inflammatory diseases. In systemic lupus erythematosus (SLE) and rheumatoid arthritis (RA), the A allele is associated with protection and milder disease, likely by modulating VEGF expression and inflammation [59–62]. It is also more frequent in women with recurrent implantation failure, suggesting impaired angiogenesis [63], and has been linked to autoimmune thyroid disease severity [64]. While not directly linked to MS, these conditions share VEGF-related inflammatory and immune pathways relevant to MS pathogenesis. Rs1570360 has also been studied in various vascular and inflammatory diseases, including cancers, age-related macular degeneration (AMD) and cardiovascular disorders [65–79]. In addition to that, the rs699947 polymorphism also influences VEGF expression and is linked to autoimmune, inflammatory, vascular and ocular diseases. In a Spanish cohort, the AA genotype and A allele were associated with reduced risk of late-onset Alzheimer’s disease, especially in those without the APOEε4 allele [80]. Rs699947 affects rheumatoid arthritis susceptibility, with the A allele increasing risk and the C allele being protective [81,82]. It is implicated in Kawasaki disease and coronary artery lesions through angiogenesis and inflammation [83]. In ocular diseases, the C allele correlates with higher VEGF-A levels in exudative AMD [68,69] and is associated with diabetic retinopathy risk across populations [84,85]. Beyond these, rs699947 has been studied in cardiovascular diseases, cancers and neuropsychiatric disorders, highlighting its broad role in VEGF-related vascular and immune regulation with potential relevance to MS [86–88]. Similarly, rs3025033 has been mainly studied in AMD, where it promotes retinal endothelial cell proliferation without affecting the anti-angiogenic VEGF165b isoform, suggesting a role in pathological neovascularization [89]. Although it shows genotype differences between wet and dry AMD subtypes, its overall association with AMD susceptibility is unclear [90]. It has also been linked to bladder cancer risk, reflecting its role in angiogenesis-related diseases [91]. These findings suggest rs3025033 influences VEGFA-driven cell proliferation and may affect disease phenotypes *via* alternative splicing and vascular remodeling. Moving to rs2146323, this polymorphism affects VEGF expression and brain structure; the A allele is linked to reduced hippocampal volume and lower psychosis risk [92,93]. It also influences asthma treatment response, with the AA genotype showing better corticosteroid outcomes but worse control with leukotriene antagonists [94], possibly linking shared inflammatory pathways in MS. Rs2146323 is associated with diabetic retinopathy, AMD and ovarian cancer survival, reflecting its role in vascular remodeling and angiogenesis [95–98]. Additional studies support its involvement in AMD and ovarian cancer outcomes [91,99–102]. The rs1413711 polymorphism has been linked to AMD subtypes and severity, with the A allele increasing risk especially for dry AMD in a Turkish population [90]. It is also associated with optic neuritis development, a condition related to MS, suggesting involvement in neuroinflammation [103]. Beyond neurovascular diseases, rs1413711 has been studied in ovarian cancer and intracerebral haemorrhage, indicating broader effects on vascular and inflammatory conditions [104,105]. Finally, rs833061 influences VEGF expression and angiogenesis, processes crucial for MS pathogenesis *via* neuroinflammation and blood-brain barrier integrity. It is linked to altered risk and treatment outcomes in AMD [106,107], impacts survival in non-small-cell lung cancer [65] and affects clinical response in gastrointestinal stromal tumours [108]. Additionally, rs833061 is associated with diabetic retinopathy and breast cancer susceptibility, highlighting its broad impact on vascular and inflammatory pathways [73,109].

## 5. Conclusions

In conclusion, this study evaluated the distribution of *VEGFA* polymorphisms and circulating VEGFA levels in patients with multiple sclerosis. Although several nominal associations between VEGFA variants and disease-related measures were observed, none remained significant after correction for multiple testing. Therefore, these findings should be interpreted with caution. A significant difference in serum VEGFA levels between MS patients and controls was identified; however, this observation may be influenced by unmeasured confounding factors, including treatment exposure. Overall, the results suggest that VEGFA may be involved in MS-related biological processes, but the present findings are exploratory and hypothesis-generating. Further studies in larger, well-characterized cohorts, incorporating treatment data and subtype-specific analyses, are required to clarify the role of VEGFA in multiple sclerosis.

## 6. Limitations

This study has several limitations that should be considered when interpreting the results. First, although a relatively large cohort was used for genetic analyses, the serum VEGFA measurements were performed in a smaller subgroup, which limited statistical power, particularly for genotype–phenotype analyses where some genotype categories included only a few individuals. Therefore, these findings should be considered exploratory. Second, multiple testing correction using the Bonferroni method resulted in no statistically significant association**s**, indicating that the observed nominal associations may reflect chance findings and should be interpreted with caution. Third, clinical data on disease-modifying therapies were not systematically available, and thus, the potential influence of treatments such as natalizumab or anti-CD20 therapies on VEGFA levels could not be assessed. This represents an important potential confounding factor. Fourth, analyses were performed using a combined MS cohort without stratification by disease subtype (e.g. RRMS vs. SPMS) due to limited sample size in progressive MS groups, which may obscure subtype-specific effects. Fifth, age stratification was based on the cohort median rather than a biologically defined threshold, and therefore, age-related findings should be considered exploratory. In addition, dichotomization of age may reduce statistical sensitivity. Finally, the study population consisted of individuals from a single geographic region, which may limit the generalizability of the findings to other populations. Taken together, these limitations indicate that the results should be interpreted as hypothesis-generating and further studies with larger, well-characterized cohorts, detailed treatment data and subtype-specific analyses are required to validate these findings.

## Supplementary Material

Supplementary material.docx

## Data Availability

The datasets used and/or analysed during the current study are available from the corresponding author upon request.
