# Supplementary material for: Sex- and age-specific associations of VEGFA polymorphisms with multiple sclerosis susceptibility
Source: Ann Med. 2026 Aug 22;58(1):2715843. doi: 10.1080/07853890.2026.2715843 (PMC13502499; doi:10.1080/07853890.2026.2715843)
Supplement: Supplementary file 1 [file IANN_A_2715843_SM8200.docx]

# **Supplementary material**

# **Table S1. Hardy–Weinberg Equilibrium in the Control Group**

| SNP | Chi-square (χ²) | HWE p-value | HWE status |
| --- | --- | --- | --- |
| *VEGFA* rs1570360 | 16.04 | 0.0001 | Deviates from HWE |
| *VEGFA* rs699947 | 0.67 | 0.414 | In HWE |
| *VEGFA* rs3025033 | 4.28 | 0.039 | Deviates from HWE |
| *VEGFA* rs2146323 | 1.34 | 0.248 | In HWE |
| *VEGFA* rs1413711 | 2.62 | 0.106 | In HWE |
| *VEGFA* rs833061 | 2.62 | 0.106 | In HWE |

Note. Hardy–Weinberg equilibrium was assessed in the control group only.
